# Potential ectoparasiticide for dog and cat fleas, a combination of *Ficus minahassae* extract and latex from *Carica papaya* L.

**DOI:** 10.5455/javar.2024.k833

**Published:** 2024-09-30

**Authors:** Dina Victoria Rombot, Yermia Semuel Mokosuli

**Affiliations:** 1 Kampus Fakultas Kedokteran Malalayang, Manado, Sulawesi Utara, Indonesia; 2 Kampus Unima Tonsaru Tondano Sulawesi Utara, Indonesia

**Keywords:** *Ctenocephalides* sp., *Ficus minahassae*, latex *Carica papaya*, toxicity, LD_50_

## Abstract

**Objective:**

This study aimed to analyze the compound content of the combined extracts of Langusei fruit (*Ficus minahassae* L.) and papaya latex (*Carica papaya* L.) and perform bioassays of the combination of extracts on fleas of the genus *Ctenocephalides*.

**Materials and Methods:**

Liquid chromatography-tandem mass spectrometry (LC-MS/MS) determined the chemical composition of the combined extract. The toxicity of the extract combination was evaluated *in vivo* on cat flea and dog flea imago separately. The combination of phytochemical screening of Langusei extract and papaya latex contained all the main phytochemical compounds.

**Results:**

The LCMS/MS analysis results showed that the combination of Langusei extract and papaya latex contained six compounds. Four compounds were identified: 3-butenyl glucosinolate, erythromycin, aluminum palmitate, and henpentakontilbenzene. Four compounds indicate a new compound. The toxicity of the combined extract was highest for both *Ctenocephalides felis* and *Ctenocephalides canis* in the P4 treatment (10%), with an average mortality of 100%, the same as the control mortality of the synthetic insecticide deltamethrin. The best LD_50_ for *C. felis* was in the F1 formula (4.003 mg/l), while in *C. canis* it was shown in the F3 (3.733 mg/l).

**Conclusion:**

Based on the results obtained, the combination of Langusei fruit extract and papaya latex can be developed as an ectoparasiticide for fleas of the genus *Ctenocephalides*.

## Introduction

Cat and dog fleas (*Ctenocephalides*) are the most important ectoparasites found in dogs and cats worldwide. *Ctenocephalides* sp. is a mandatory hematophagous ectoparasite of wildlife, domestic cats, and dogs worldwide. *Ctenocephalides* have a high reproductive rate, making them a significant health problem in dogs and cats [1,2]. As animals are mostly cared for by humans, dogs and cats have close contact with humans daily. *Ctenocephalides felis* and *Ctenocephalides canis* are the most common fleas, infecting not just dogs and cats but also other warm-blooded animals, including humans [3]*. Ctenocephalides felis*, in particular, is the most common species worldwide, with significant infestation rates. Because of their excellent resilience to a wide range of climatic circumstances, they have a cosmopolitan distribution [4]. *Ctenocephalides* sp. can cause allergic dermatitis and transmit various pathogenic bacteria to humans. The metagenomic analysis carried out in previous studies showed that many bacterial species associated with *Ctenocephalides *sp. have never been reported [5,6].

Chemical insecticides are widely used to eradicate *Ctenocephalides* sp. in dogs and cats. It was reported that resistance to cypionate and permethrin insecticides was reported in *Ctenocephalides* sp. Resistance also occurs to pyrethroid-class insecticides [7,8]. Fipronil-based pesticides should be examined for use on dog and cat fleas, according to research conducted in the United Kingdom [9]. Utilization of a combination of plant extracts is widely used to control paint fleas. The combination of cassia, thyme, and oregano essential oils has an insecticidal effect on adult cat flea stages [10]. On cat and dog fleas, essential oils from *Illicium verum* and *Pelargonium graveolens* had insecticidal efficacy greater than 70%. [11]. In *in vitro* studies, the essential oil of *Ocimum gratissimum* was effective against all stages of ticks, producing adulticide (LC50 = 5.85 gm cm^2^), ovicidal (LC50 = 1.79 gm cm^2^), and larvicidal (LC50 = 1.21 gm cm^2^) mortality at low concentrations [12]. *Cannabis sativa* essential oil exhibited insecticidal activity (100% mortality at the highest concentration) for flea control at egg, larval, pupal, and adult stages, with an LC50 of 32.45 μg/cm^2^ [13].

The insecticidal potency of the combination of papaya latex extract and *Ficus minahassae* fruit has never been reported. Papaya latex contains many secondary metabolites, such as alkaloids, steroids, saponins, and proteases, which have insecticidal activity [14,15]. Papaya latex has vigorous insecticidal activity on *Rhipicephalus microplus* and *Aedes aegypti* [16]. In contrast, Langusei (*F. minahassae*) has been used empirically by the Minahasa community as a raw material for botanical insecticides. However, the analysis of the bioactive content of the combination of papaya latex extract and *F. minahassae* has never been reported. Combining plant extracts and papaya latex can increase insect toxicity [14,17]. Furthermore, bioassays on insects, especially on dog and cat fleas, have not been reported. Research has been carried out to analyze the compound content of papaya latex extract and *F. minahassae* fruit and to conduct bioassays against ticks of the genus *Ctenocephalides.*

## Materials and Methods

### Plant sample collection

Langusei fruit (*F. minahassae*) was obtained from the forest of Mount Klabat, North Minahasa district. In contrast, latex papaya (*Carica papaya*) was obtained from a local papaya plantation in Matungkas, Dimembe District, North Minahasa Regency, North Sulawesi. Langusei fruit was determined in the Biology Laboratory of the Faculty of Mathematics, Natural Sciences, and Earth Sciences, Manado State University. Papaya latex used is papaya fruit latex and preserved in sterile sample bottles, stored in a box with a temperature of 25°C.

### Cat and dog flea sample collection

*Ctenocephalides felis* and *C. canis* were obtained from North Minahasa Regency, Tomohon City, and Minahasa Regency, Sulawesi Utara, Indonesia. The total sample of cats that were the source of fleas for each region was 50 adult individuals. The total sample of dogs that were the source of ticks for each region was 50. Cat fleas and dog fleas were preserved alive and immediately brought to the laboratory for bioassays.

### Extraction and phytochemical screening

Langusei fruit is extracted in the form of wet simplicia. Simplisia preparation involves utilizing a fine blender. The simplicia was macerated using 90% alcohol (Kimia Farma). Comparison of simplicia and solvent 1:4 (w/v). This study determined that 250 gm of simplicia were macerated with 1,000 ml of 90% alcohol in a sterile glass container. Maceration was carried out at room temperature, stirring every hour to maximize the extraction process. Maceration was carried out for 72 h. One day before filtering, the macerate was placed in the Mammert incubator at 45 rpm. Then, the mixture was filtered using Whatman 41 filter paper, the filtrate was separated, and the dregs were followed by maceration again according to the previous procedure. The filtrate was then evaporated using a Heidolph rotary evaporator at 45°C and 50 rpm until a semisolid and concentrated crude extract was obtained. Screening for phytochemical groups used the Harborne method. The intensity of the content has been assessed by comparing the color and precipitate generated to the control [17].

### Analysis of compound content with LC-MS/MS

The compound content of the combination of Langusei fruit extract and papaya latex was analyzed using the liquid chromatography-tandem mass spectrometry (LC-MS/MS) method [18,19]. The combination of Langusei extract and papaya latex (1.4 mg) was dissolved in 100 ml methanol. The solution was filtered using a 0.2 μm GHP filter and injected into the ultra-performance liquid chromatography system. The LC-MS system used is Xevo-ToF-1, which is equipped with a C-18 column (particle dimensions 1.8 μm, 2.1 × 100 mm^2^), MS with Xevo G2-S resolution, acquisition mode QTOF ESI (-), and MSE. The eluent consisted of 0.1% formic acid in distilled water (A) and 0.1% formic acid in acetonitrile (B). The total running time is 20 min at 100°C. The elution system was run with gradient elution at 0–1 min, the ratio of solvent A was 70%, and solvent B was 30%; at 6–18 min, solvent A was 5% solvent, B was 95%, and at 19–20 min, the solvent is a linear gradient elution: A 70% solvent, B 30%. Furthermore, data processing is performed using MassLynx software. The findings of the LC/MS-MS data analysis are shown as chromatograms, which are plots of the peak height and molecular weight of the compounds in the extract, allowing the number of compounds in each sample to be determined.

### Ectoparaticide bioassay

**LD**_50_ **determination**

The test solution was prepared using a combination of Langusei extract and papaya latex. Formula 1 is a ratio of Langusei extract to papaya sap of 1:1 (w/v). Formula 2 is a ratio of Langusei extract and papaya sap of 1:2 (w/v). Formula 3 is a ratio of Langusei extract and papaya sap of 1:3 (w/v). Each formula was tested on 10 *C. canis* on cotton-lined Petri dishes. The test concentrations used were 5, 15, 45, and 65 mg/l. Each test concentration was carried out in three replicates. The solution of each formula was sprayed on *C. canis* imago every 6 h for 24 h. *Ctenocephalides canis* imago is declared dead if there is no movement response after being given a stimulus or a gentle touch using a pin. The same method was applied to *C. felis*.

### Ectoparasiticide test

Preparation of the test solution utilizing a combination of previously prepared extracts. Three groups of test solutions were made. Preparing the test solution used Tween 80.2% (v/v) solvent to increase the solubility of the extract. Tween 80.2%, 2 ml added with 98 ml of distilled water. A 2.5% (w/v) concentration test solution was prepared by weighing 2.5 gm of each combination of extracts (F1, F2, and F3) and then putting it into a volumetric flask and adding Tween 80 solvent (2%) until the volume reached 100 ml. The same steps were carried out to prepare the extract solution with a test concentration of 5% (w/v) and 10% (w/v). The test solution, which has been labeled for research, is put into each sprayer.

During the preparation stage, the petri dish container is given a cotton pad to cover it uniformly at the bottom, then sprayed evenly with each test solution. The spraying was done three times. Furthermore, each *C. felis* and *C. canis* imago were divided into four treatment groups with three replications. Each petri dish contains ten fleas. They were spraying each group, namely P0 as a control with 2% Tween 80 solvent, P1 as a positive control with deltamethrin 0.5EC (Butox 50^®^) concentration of 0.05%, and groups P2, P3, and P4 sequentially with ethanol extract of each plant concentrations of 2.5%, 5%, and 10%. After being treated, observations were made on the number of dead fleas based on the predetermined observation time. In general, fleas will always move actively on the Petri dish, and fleas are declared dead if there is no response to movement after being given a stimulus or a gentle touch using a pin. Observations to see the number of fleas killed were carried out at 1 minute (shortly after treatment until the first 1 min), 15, 30, and 60. The number of dead fleas was determined as the death rate due to the insecticidal activity of the extract combination. The same procedure was carried out on *C. felis*.

### Data analysis

Data from LC-MS/MS analysis are interpreted descriptively. The molecular weight of the LC-MS/MS output was used to search for the most similar compound in two online organic compound databases, namely the National Institute of Standards and Technology (NIST) (https://webbook.nist.gov) USA and Advanced Industrial Science and Technology (AIST) (https://sdbs.db.aist.go.jp) Japan. Data on the mortality of ticks from the genus *Ctenocephalides* were analyzed by variance, followed by the Duncan multiple range test (DMRT) if there were significant differences between treatments. Mortality data was used to determine lethal dose 50 (LD_50_) by probit analysis. Statistical analysis using IBM SPSS Program 25.

## Results and Discussion

### Combination of extracts

The ethanol extract of Langusei fruit has a blackish-brown color. The weight of the extract obtained was 33.8 gm, with a yield of 12.6%. The aroma of Langusei fruit extract. Papaya sap is milky white with a distinctive papaya aroma. Papaya latex is tapped from young papaya fruit, preserved in sample bottles, and stored in the refrigerator at 25°C (Fig. 2). Langusei‘s ethanol extract is then combined with papaya latex. Phytochemical screening showed that the combination of extracts contained all the main phytochemical groups. However, based on the intensity of the color and precipitate, the ethanol extract of Langusei contains alkaloids, flavonoids, saponins, and tannins at a higher intensity than steroids and terpenoids. Phytochemical screening on the combination of Langusei extract and papaya latex showed increasing intensity differences in steroid and triterpenoid content. On the other hand, the content of saponins and tannins decreased compared to before being mixed with papaya latex (Table 1).

### LC-MS/MS analysis of a combination Langusei extract and latex papaya

**LC analysis**

**Figure 1.**
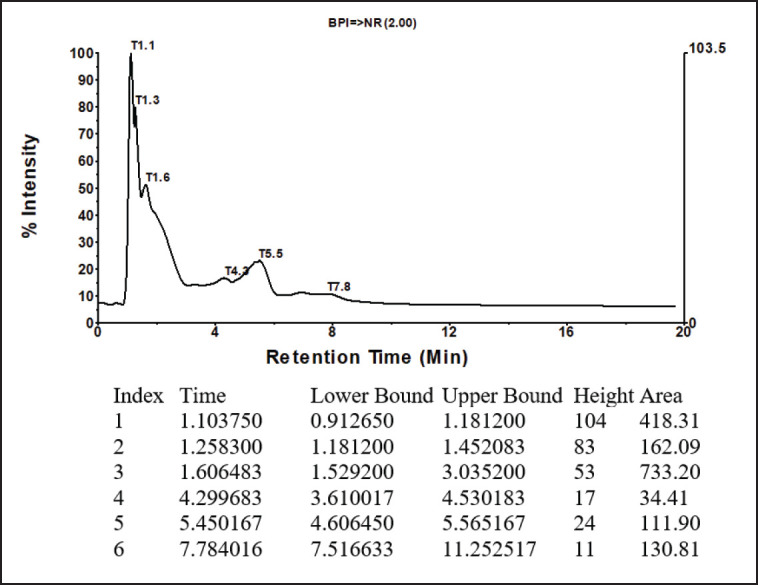
LC profile mixture of langusei extract and papaya sap.

According to the LC results, the mixture of Langusei extract and papaya latex consists of six compounds, which can be seen at retention numbers of 1.103, 1.258, 1.606, 4.299, 5, 450, and 7.784 m. The compound with the highest retention was 1.60, subsequent to 1.103, while the compound with the lowest retention was 4,299 (Fig. 1). Chemical substances isolated by LC are then evaluated using MS.

### MS analysis

**Retention compound 1.103**

As shown in the graphic below, compounds having a retention of 1.103 exhibit fractionation. According to the foregoing findings, a molecule with a retention of 1.457 has a molecular weight of 733.76 (Table 2). A search for molecular weight data on the NIST Chemistry WebBook SRD 69 obtained a similar compound based on molecular weight, namely 3-butenyl glucosinolate, TMS (C26H59NO9S2Si5).

A search for compounds based on molecular weight at https://sdbs.db.aist.go.jp/sdbs/cgi-bin/direct_frame_top.cgi found that the closest compound is (-)-erythromycin (C37H67NO13) with molecular weight 733.9 (Table 2).

**Compound retention: 1.1258**

As is apparent in the graphics below, compounds with a retention of 1.1258 generate fractionation. According to these findings, a molecule with a retention of 1.60 has a molecular weight of 733.76 (Table 2). Searching data for molecular weights that are similar to compounds with a retention of 1.25 obtained 3 compounds (NIST Chemistry WebBook SRD 69). The most similar compound based on molecular weight is erythromycin (C37H67NO13).

**Retention compound 1.606**

According to the data in the graphic below, compounds with a retention of 1.606 generate fractionation. Table 2 shows that the molecular weight of the molecule with a retention of 1.606 is 1158.18. The NIST Chemistry WebBook SRD 69 contains no search results for a molecular weight similar to a molecule with a retention of 5.89. This is thought to be a novel chemical.

**Retention compound 4.299**

As is evident in the graphic below, compounds with a retention of 4.299 generate fractionation. The molecular weight of the molecule with a retention of 4.299 is 1148.56 (Table 2). The NIST Chemistry WebBook SRD 69 contains no search results for a molecular weight similar to a molecule with a retention of 4.299. The chemical is believed to be novel.

**Retention compound: 5.450**

As demonstrated in the graphic below, compounds with a retention of 5.450 generate fractionation. Table 2 shows that the molecular weight of the molecule with a retention of 5.450 is 1150.17. The NIST Chemistry WebBook SRD 69 does not include any search data for a molecular weight similar to a molecule with a retention of 5.450. It is believed that the chemical is novel.

**Retention compound 7.784**

As demonstrated in the graphic below, compounds with a retention of 7.784 generate fractionation. Table 2 shows that the molecular weight of the molecule with a retention of 7.784 is 793.00. A search for molecular weight data on the NIST Chemistry WebBook SRD 69 obtained two similar compounds, namely aluminum palmitate (C48H93AlO6) with a molecular weight of 793.20 and henpentacontylbenzene (C57H108) with a molecular weight of 793.46.

**Figure 2.**
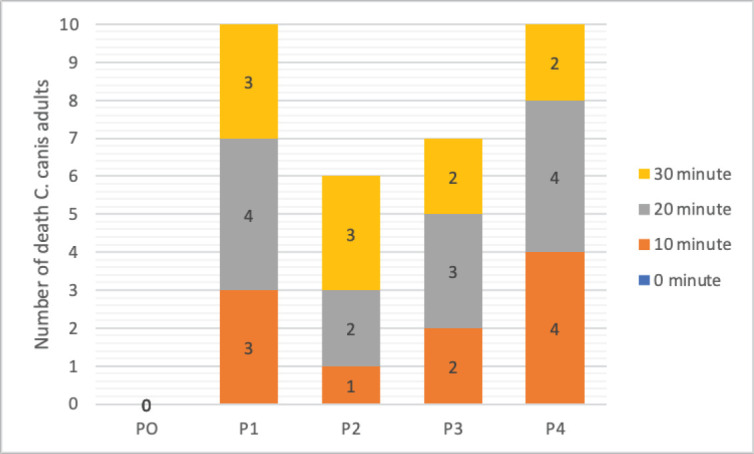
The trend of *C. canis* mortality after combination extract treatment compared to control.

**Table 1.** Results extraction and phytochemical screening.

No.	Samples	Extract weight (gm)	% yield	Phytochemical screening						Method
				A	F	S	T	St	Tr	
1	Langusei extract	33.8	12.6	++	++	++	++	+	+	Harborne method
2	Combination of langusei extract and latex of papaya	Ratio 1:1 (w/v)		++	++	+	+	++	++	Harborne method

*Description:* A: Alkaloids, F: Flavonoids, S: Saponins, T: Tannins, St: Steroids, Tr: Triterpenoids. +: indicates the intensity of the content based on color and precipitate

**Table 2.** Compound retention time and MS profile.

No.	Compound retention time	MS profile
1	1.103	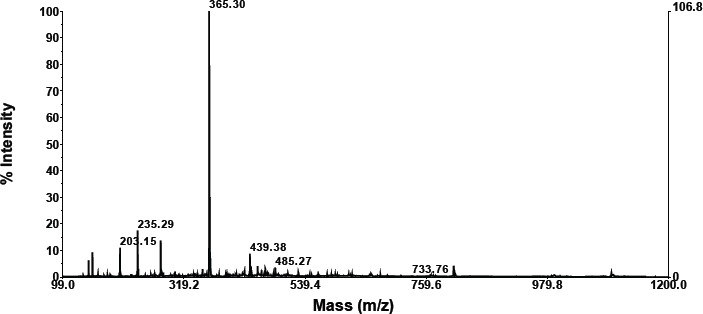
2	1.128	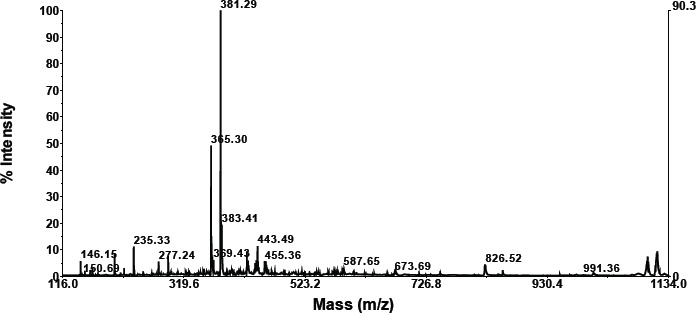
3	1.606	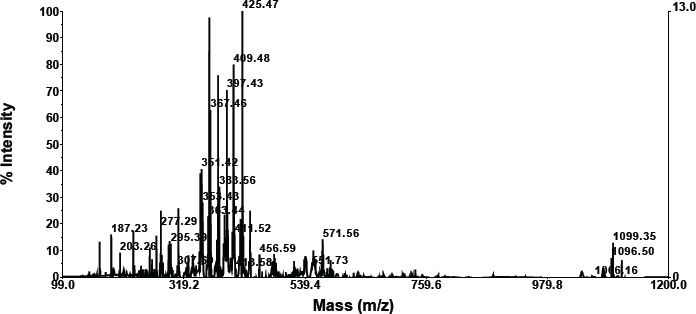
4	4.299	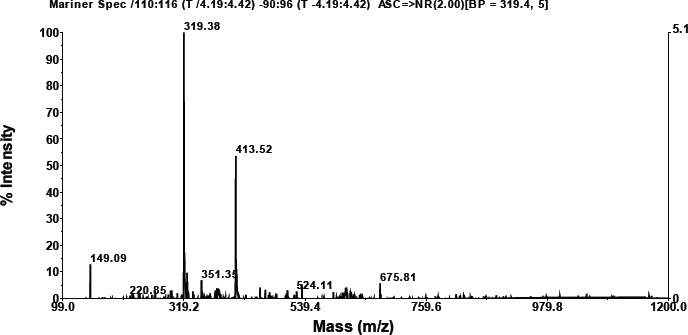
5	5.450	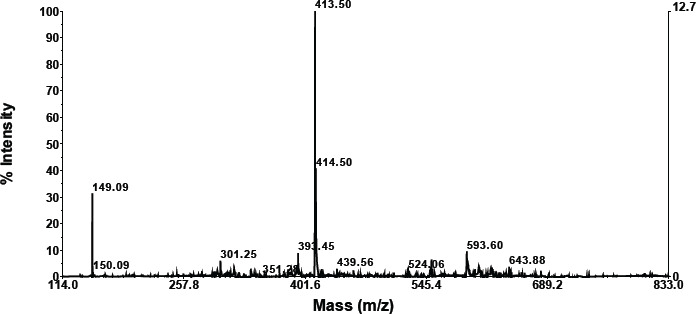
6	7.784	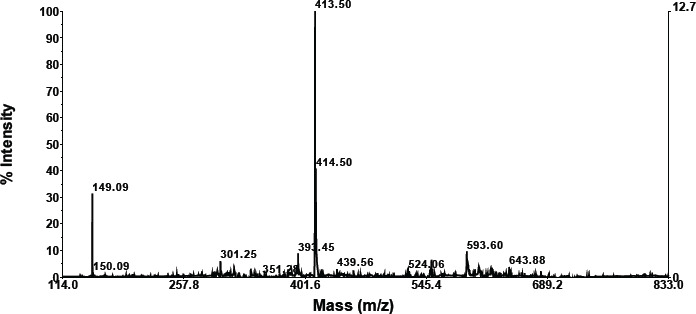

### Ectoparaticide bioassay

A total of 10 *C. canis* individuals tested were adults. At minute zero, all treatments and controls had no death of *C. canis*. In the 10th minute, the highest mortality was shown in the P4 and P1 positive controls. In the 20th minute, half of the test animal population had died, as shown in the P3, P4, and control P1 treatments. In the P2 and P3 treatments, only eight individual *C. canis* died after 30 min of treatment. However, in the P4 and P1 treatments, all *C. canis *died (Fig. 2).

**Figure 3.**
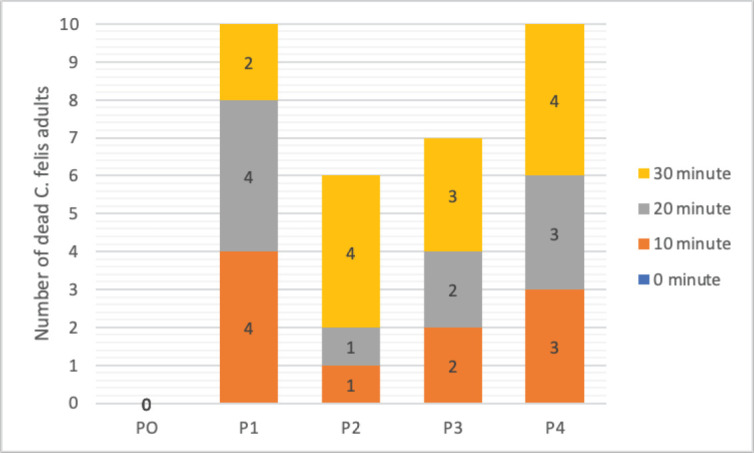
The trend of *C. felis* mortality after combination extract treatment compared to control.

A total of 10 *C. felis* individuals tested were adults. At minute zero, all treatments and controls had no death of *C. felis*. At 10 and 20 min, the highest mortality was shown in P1, 4 individuals, and P4, 3 individuals (Fig. 3). In the P2 and P3 treatments, only 6 and 7 individual *C. felis* died after 30 min of treatment. However, in the P4 and P1 treatments, all *C. felis* died (4).

### Analysis of variance

The analysis of variance of the five treatment combinations of extracts on *C. canis* showed a significant difference, where Fcount was 1.887 with a significance of 0.165 (*α* = 0.05). In the DMRT follow-up test, only P1 showed differences between treatments with P0, P2, P3, and P4. The analysis of variance of the five treatment combinations of extracts on *C. felis* canis showed a significant difference, where Fcount was 0.694 with a significance of 0.607 (*α* = 0.05). Duncan’s further test found no significant differences between treatments (*α* = 0.05) between P0, P1, P2, P3, and P4.

### Lethal dose 50 (LD_50_)

**Imago** ***C. felis***

**Figure 4.**
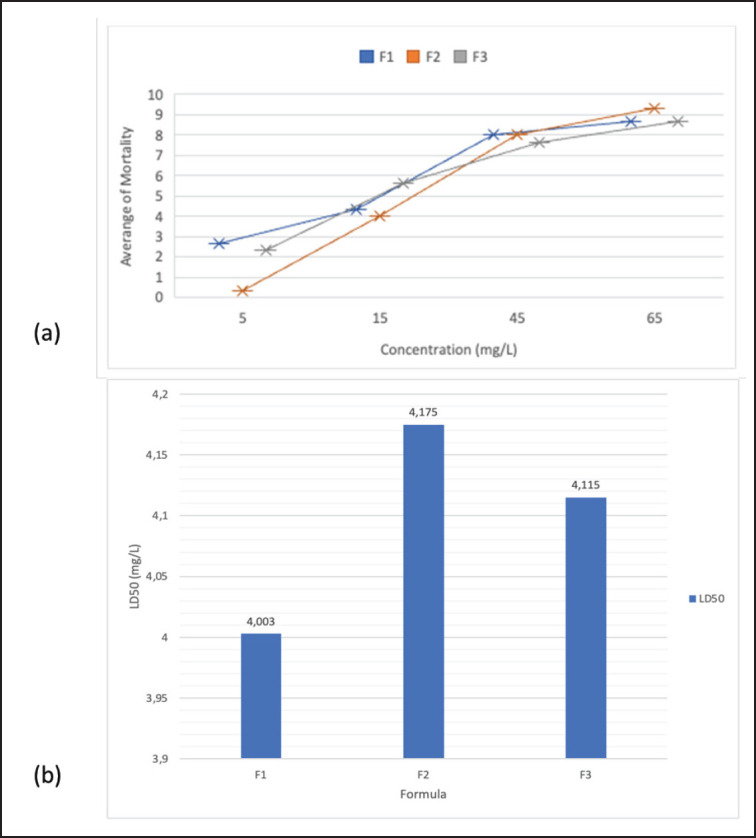
(a) The mortality rate of combination extract formulations in *C. felis*. (b) LD_50 _extract against *C. felis.*

The highest *C. felis* mortality after exposure to combined extract solutions was shown at a test concentration of 65 mg/l for all formulas. The lowest mortality rate was shown at the test concentration of 5 mg/l for all formulas. The formula with the highest average mortality is shown in F2, which is 9.33 (Fig. 4). Mortality data was used to determine the LD_50_ using probit analysis with the SPSS program. The results of the probit analysis showed the highest toxicity in F1 (LD_50_ = 4.003 mg/l) and the lowest toxicity in F2 (LD_50_ = 4.175 mg/l) (Fig. 4).

**Figure 5.**
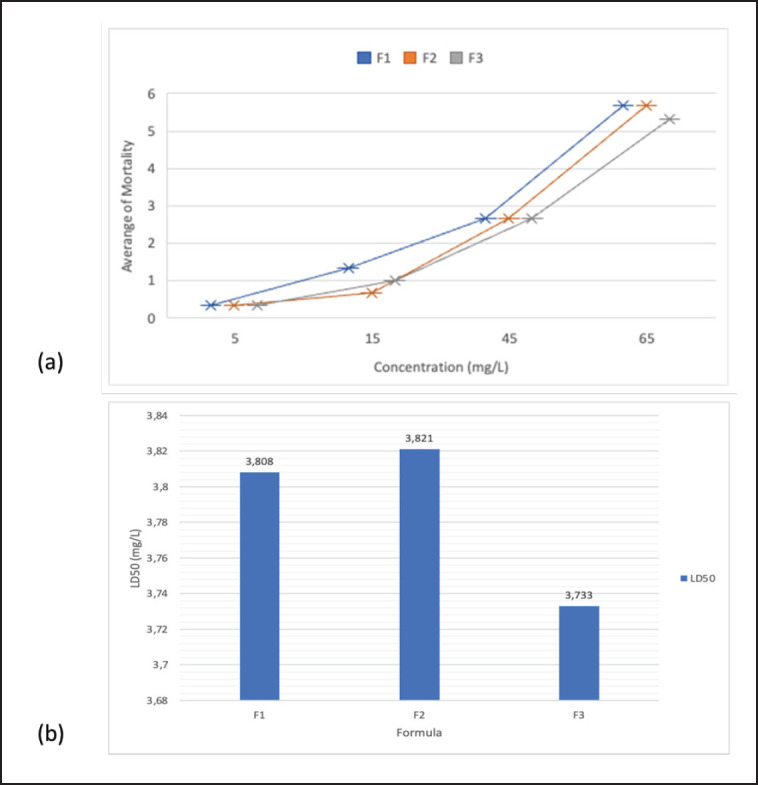
(a) The mortality rate of combination extract formulations in *C. canis*. (b) LD50 combination of F1, F2, and F3 extracts against *C. canis.*

**Imago** ***C. canis***

The highest *C. canis* mortality after exposure to combined extract solutions was shown at a test concentration of 65 mg/l for all formulas. All formulas had the lowest mortality rate at 5% test concentration. The combination of extracts with the highest average mortality is shown in F1, which is 8.33 (Fig. 5). Mortality data is used to determine the LD_50_ using probit analysis in the SPSS program. The results of the probit analysis showed the highest toxicity in Formula 3 (LD_50_ = 3.733 mg/l) and the lowest toxicity in Formula 2 (LD_50_ = 3.823 mg/l) (Fig. 5).

Based on the mortality test, the combined extract formula showed a different level of toxicity in *C. felis* and *C. canis*. F2 showed the highest toxicity activity on *C. felis*, while F1 showed the most increased toxicity on *C. canis*.

## Discussion

Phytochemical screening showed that Langusei extract contained alkaloids, flavonoids, saponins, and tannins in high intensity, while triterpenoids and steroids were in moderate intensity. Papaya latex is rich in proteases but also includes peptides, other proteins, and the main phytochemical groups [20,21]. The phytochemical screening of the combination of Langusei ethanol extract and papaya latex showed high levels of alkaloids, flavonoids, triterpenoids, and steroids, while saponins and tannins had moderate intensity. The content of proteases in papaya latex degrades phytochemical compounds that contain peptides and protein elements [22,23]. This causes a change in the intensity of the content of the phytochemical group in the Langusei extract after being combined with papaya latex.

The combination of Langusei fruit extract and papaya latex was successfully analyzed using LC-MS/MS. LC-MS/MS is a high-resolution analytical technique and can be used in quantitative and structural analysis to provide a beneficial approach to determining the profile of a metabolite [24,25]. The molecular weight, structure, identity, and quantity of individual sample components can be determined using LC-MS/MS data. Compounds are separated based on their respective interactions with the particle’s chemical layer (stationary phase) and solvent elution via the column (mobile phase) [26]. The advantage of LC-MS is that it can evaluate a wider range of components, including thermally labile, high polarity, and high molecular mass chemicals, as well as proteins. The combination of ethanol extract from *F. minahassae* fruit and papaya latex produced five compounds based on LC-MS/MS analysis. Of the five compounds detected based on retention and molecular weight, only two were successfully identified based on searching the online organic compound database. Thus, three compounds have not been reported, so they are stored in the NIST (USA) and AIST (Japan) databases. The two compounds have a molecular weight of 1158.182, respectively, with a retention of 2.54, and 880.61, with a retention of 8.29. The two compounds are thought to be new compounds.

The ectoparasiticide test showed the highest average mortality in the P4 treatment for *C. canis* and *C. felis*. Based on the variance analysis, the extract combination treatment affected the mortality of *C. canis* and *C. felis* (*p* < 0.05). Even though the DMRT test did not show any differences between treatments, the compound content in the combination of extracts can have a toxic effect on *C. canis* and *C. felis*. Compounds detected in LC-MS play a role in the toxicity that causes death in *C. canis* and *C. felis*. The compounds identified as 3-butenyl glucosinolate, TMS (C26H59NO9S2Si5), erythromycin (C37H67NO13), aluminum palmitate (C48H93AlO6), and henpentakontilbenzene (C57H108) menyebabkan iritasi [27–29].

The combination extract formulation 1 (F1) showed the highest toxicity with an LD_50_ of 4.003 mg/l compared to F1 (4.175 mg/l) and F3 (4.115 mg/l) for testing on *C. felis*. The best LD_50_ for *C. canis* is F3 (3.733 mg/l), followed by F1 (3.808 mg/l) and F3 (3.821 mg/l). Thus, the LD_50_ of the extract combination has a different effect on *C. canis* and *C*.* felis*. In *C. felis*, the composition of papaya latex, which was more in the combination of extracts, gave a strong toxic effect. In contrast, in *C. felis,* the same ratio of *F. minahassae* extract and papaya latex showed a strong toxic effect. Cysteine protease in papaya latex is a natural protector against insect pests on papaya fruit [14,30–32]. Papaya latex causes acute toxicity to *A. aegypti*, *Culex quinquefasciatus*, and *Sitophilus zeamais* [16,33–35]. The proteolytic activity of papaya latex damages the cuticle of *R. microplus* [36]. Papaya latex contains proteases and secondary metabolite compounds such as alkaloids, terpenoids, proteins, phenols, and phytochemicals [15,30]. Papaya latex is toxic to insects, molluscs, and fungi. The high LD_50_ explains that the combination of *F. minahasae* fruit extract and papaya latex is synergistic in toxicity to the tested insects. As a Minahasa endemic plant, *F. minahassae* has little reported use as an insecticide. The high content of phenolic compounds in *F. minahassae* fruit extract is consistent after being combined with papaya latex. Many phenolic compounds are reported to have insecticidal activity [36]. Allelochemicals from plants *Ficus* sp., *F. benghalensis*, and *F. religiosa* are insecticidal in insects in a broad spectrum [37,38]. *F. minahasae* is reported to have antibacterial activity [39]. The imago *Ctenocephalides* sp. was exclusively examined in this study. Combinations of extracts should be tested on different life stages of *Ctenocephalides* in the immature phase in the future.

## Conclusion

The combination of phytochemical screening of *F. minahassae* extract and papaya latex contained all the main phytochemical compounds but showed high intensity for compounds belonging to the alkaloid, flavonoid, triterpenoid, and steroid groups. Based on LCMS/MS analysis, the combination of *F. minahassae* extract and papaya latex contained five compounds with retention: 1.103, 1.258, 1.606, 4.299, 5, 450, and 7.784 m. Four compounds were identified: 3-butenyl glucosinolate, TMS (C26H59NO9S2Si5), erythromycin (C37H67NO13), aluminum palmitate (C48H93AlO6), and henpentakontilbenzene (C57H108). Compounds with retentions of 1.60 (1158.18); 4.299 (1148.56); and 5.450 (1150.17) have not been recorded in the international organic compound database, thus indicating a new compound. The toxicity of the combination extract was highest for both *C. felis* and *C. canis* in the P4 treatment (10%) with an average mortality of 100%, the same as the control mortality of the synthetic insecticide deltamethrin. The best LD_50_ for *C. felis* was in the F1 formula (4.003 mg/l), while in *C. canis* it was shown in the F3 formula (3.733 mg/l).

## List of Abbreviations

AIST, Advanced Industrial Science and Technology; DMRT, Duncan multiple range test; LC-MS/MS, liquid chromatography tandem mass spectrometry; LD_50_, lethal dose 50; NIST, National Institute of Standards and Technology.
